# A Partial Information Decomposition for Multivariate Gaussian Systems Based on Information Geometry

**DOI:** 10.3390/e26070542

**Published:** 2024-06-25

**Authors:** Jim W. Kay

**Affiliations:** School of Mathematics and Statistics, University of Glasgow, Glasgow G12 8QQ, UK; jim.kay@glasgow.ac.uk

**Keywords:** partial information decomposition, mutual information, synergy, redundancy, information geometry

## Abstract

There is much interest in the topic of partial information decomposition, both in developing new algorithms and in developing applications. An algorithm, based on standard results from information geometry, was recently proposed by Niu and Quinn (2019). They considered the case of three scalar random variables from an exponential family, including both discrete distributions and a trivariate Gaussian distribution. The purpose of this article is to extend their work to the general case of multivariate Gaussian systems having vector inputs and a vector output. By making use of standard results from information geometry, explicit expressions are derived for the components of the partial information decomposition for this system. These expressions depend on a real-valued parameter which is determined by performing a simple constrained convex optimisation. Furthermore, it is proved that the theoretical properties of non-negativity, self-redundancy, symmetry and monotonicity, which were proposed by Williams and Beer (2010), are valid for the decomposition $I_{\mathrm{ig}}$ derived herein. Application of these results to real and simulated data show that the $I_{\mathrm{ig}}$ algorithm does produce the results expected when clear expectations are available, although in some scenarios, it can overestimate the level of the synergy and shared information components of the decomposition, and correspondingly underestimate the levels of unique information. Comparisons of the $I_{\mathrm{ig}}$ and $I_{\mathrm{dep}}$ (Kay and Ince, 2018) methods show that they can both produce very similar results, but interesting differences are provided. The same may be said about comparisons between the $I_{\mathrm{ig}}$ and $I_{\mathrm{mmi}}$ (Barrett, 2015) methods.

## 1. Introduction

Williams and Beer [1] introduced a new method for the decomposition of information in a probabilistic system termed *partial information decomposition* (PID). This allows the joint mutual information between a number of input sources and a target (output) to be decomposed into components which quantify different aspects of the transmitted information in the system. These are the unique information that each source conveys about the target; the shared information that all sources possess about the target; the synergistic information that the sources in combination possess regarding the target. An additional achievement was to prove that the interaction information [2] is actually the difference between the synergy and redundancy in a system. Thus, a positive value for interaction information signifies that there is more synergy than redundancy in the system, while a negative value indicates the opposite. The work by Willliams and Beer has led to many new methods for defining a PID, mainly for discrete probabilistic systems [3,4,5,6,7,8,9,10,11,12,13] spawning a variety of applications [14,15,16,17,18,19].

There has been considerable interest in PID methods for Gaussian systems. The case of static and dynamic Gaussian systems with two scalar sources and a scalar target was considered in [20], which applied the minimum mutual information PID, $I_{\mathrm{mmi}}$. Further insights were developed in [21] regarding synergy. A PID for Gaussian systems based on common surprisal was published in [7]. Barrett’s work [20] was extended to multivariate Gaussian systems with two vector sources and a vector target in [22] using the $I_{\mathrm{dep}}$ method which was introduced for discrete systems in [8]. Further work based on the concept of statistical deficiency is reported in [23]. Application of PID for Gaussian systems has been used in a range of applications [18,24,25,26,27,28,29,30]

We focus in particular here on the method proposed by Niu and Quinn [3]. They applied standard results from information geometry [31,32,33] in order to define a PID for three scalar random variables which follow an exponential family distribution, including a trivariate Gaussian distribution.

Here, we extend this work in two ways: (a) we provide general formulae for a PID involving multivariate Gaussian systems which have two vector sources and a vector target by making use of the same standard methods from information geometry as in [3] and (b) we prove that the Williams–Beer properties of non-negativity, self-redundancy, symmetry and monotonicity are valid for this PID. We also provide some illustrations of the resulting algorithm using real and simulated data. The PID developed herein is based on some of the probability models in the same partially ordered lattice on which the $I_{\mathrm{dep}}$ algorithm is based. Therefore, we also compare the results obtained with those obtained by using the $I_{\mathrm{dep}}$ method. The $I_{\mathrm{ig}}$ results are also compared with those obtained using the $I_{\mathrm{mmi}}$ algorithm.

## 2. Methods

### 2.1. Notation

A generic ‘*p*’ will be used to denote an absolutely continuous probability density function (pdf), with the arguments of the function signifying which distribution is intended. Bold capital letters are used to denote random vectors, with their realised values appearing in bold lowercase—so that $p(x_{1},x_{2},x_{3})$ denotes the joint pdf of the random vectors, $X_{1},X_{2},X_{3}$, while $p(x_{1},x_{3}|x_{2})$ is the conditional pdf of $\left(X_{1},X_{3}\right)$ given a value for $X_{2}$.

We consider the case where random vectors $X_{1},X_{2},X_{3}$, of dimensions $n_{1},n_{2},n_{3}$, respectively, have partitioned mean vectors equal to zero vectors of lengths $n_{1},n_{2},n_{3}$, respectively, and a conformably partitioned covariance matrix. We stack these random vectors into the random vector Z, so that Z has dimension $m=n_{1}+n_{2}+n_{3},$ and assume that Z has a positive definite multivariate Gaussian distribution with pdf $p(x_{1},x_{2},x_{3})$, mean vector 0 and covariance matrix given by

(1) $$\Sigma =\left[\begin{array}{ccc} \Sigma_{11} & \Sigma_{12} & \Sigma_{13}\\ \Sigma_{12}^{T} & \Sigma_{22} & \Sigma_{23}\\ \Sigma_{13}^{T} & \Sigma_{23}^{T} & \Sigma_{33} \end{array}\right],$$

where the covariance matrices $\Sigma_{11},\Sigma_{22},\Sigma_{33}$ of $X_{1},X_{2},X_{3}$, respectively, are of sizes $n_{1}\times n_{1},n_{2}\times n_{2},n_{3}\times n_{3}$, and $\Sigma_{12},\Sigma_{13},\Sigma_{33}$ are the pairwise cross-covariance matrices between the three vectors $X_{1},X_{2},X_{3}$. We also denote the conformably partitioned precision (or concentration) matrix *K* by

$$K=\left[\begin{array}{ccc} K_{11} & K_{12} & K_{13}\\ K_{12}^{T} & K_{22} & K_{23}\\ K_{13}^{T} & K_{23}^{T} & K_{33} \end{array}\right],$$

where $K=\Sigma^{-1}$. The pdf of Z is

(2) $$f\left(z|K\right)=\frac{\sqrt{\left|K\right|}}{{\left(2\pi \right)}^{\frac{1}{2}m}}\exp\left(-\frac{1}{2}z^{T}\;K\;z\right)\equiv \frac{\sqrt{\left|K\right|}}{{\left(2\pi \right)}^{\frac{1}{2}m}}\exp\left(-\frac{1}{2}\underset{i\leq j}{\sum_{i=1}^{3}\sum_{j=1}^{3}}x_{i}^{T}K_{ij}x_{j}\right),\;z\in R^{m}.$$

### 2.2. Some Information Geometry

We now describe some standard results from information geometry [32,33] as applied to zero-mean, partitioned multivariate Gaussian probability distributions. The fact that there is no loss of generality in making this zero-mean assumption will be justified by Lemma 1 in Section 3. The multivariate Gaussian pdf defined in (2) may be written in the form

$$\begin{array}{cc} \; & \frac{\sqrt{\left|K\right|}}{{\left(2\pi \right)}^{\frac{1}{2}m}}\exp(-\frac{1}{2}\left(x_{1}^{T}K_{11}x_{1}+x_{2}^{T}K_{22}x_{2}+x_{3}^{T}K_{33}x_{3}\right)\\ \; & \;\;\;\;\;\;-(x_{2}^{T}K_{12}^{T}x_{1}+x_{3}^{T}K_{13}^{T}x_{1}+x_{3}^{T}K_{23}^{T}x_{2})), \end{array}$$

which may be written in terms of the Frobenius inner product as

$$\begin{array}{cc} \; & \exp(\langle -\frac{1}{2}K_{11},x_{1}x_{1}^{T}\rangle +\langle -\frac{1}{2}K_{22},x_{2}x_{2}^{T}\rangle +\langle -\frac{1}{2}K_{33},x_{3}x_{3}^{T}\rangle \\ \; & \;\;\;\;\;\;+\langle -K_{12},x_{1}x_{2}^{T}\rangle +\langle -K_{13},x_{1}x_{3}^{T}\rangle +\langle -K_{23},x_{2}x_{3}^{T}\rangle -\psi \left(\theta \right)), \end{array}$$

where

$$\psi \left(\theta \right)=-\frac{1}{2}\log\frac{\left|K\right|}{{\left(2\pi \right)}^{m}}.$$

This is of exponential family form [33] (p. 34) and [34] with natural parameter

$$\theta =\{-\frac{1}{2}K_{11},-\frac{1}{2}K_{22},-\frac{1}{2}K_{33},-K_{12},-K_{13},-K_{23}\},$$

and expectation parameter $\eta =\{\eta_{ij},i=1,2,3;j=1,2,3;i\leq j\}$, where $\eta_{ij}=E\left(x_{i}x_{j}^{T}\right)=\Sigma_{ij}$. We note that there is something of a terminal ambiguity here, since a ‘parameter’ is usually a real number. It is convenient to use the more compact notation provided by matrices since this enables all of the elements of a matrix natural parameter to be set to zero simultaneously.

The exponential family distribution in (2) is a dually flat manifold [31], which we denote by *M*.

**Figure 1:**
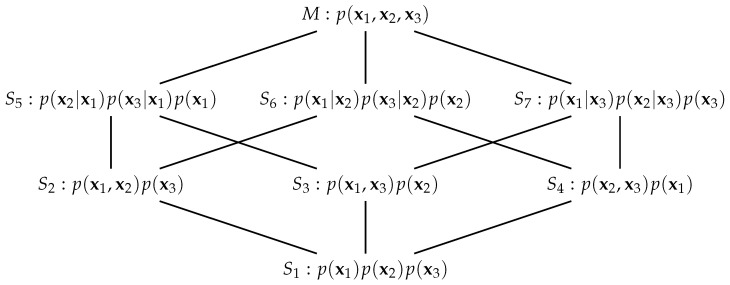
A partially ordered lattice of the manifold *M* and submanifolds $S_{1}-S_{7}$. The form of the pdf that is shown for each submanifold is that obtained by *m*-projection of the distribution $p(x_{1},x_{2},x_{3})$ onto the submanifold.

We define the following *e*-flat submanifolds of *M*:

$$\begin{array}{cc} S_{7} & =\{p\in M:K_{12}=0\}\\ S_{6} & =\{p\in M:K_{13}=0\}\\ S_{5} & =\{p\in M:K_{23}=0\}\\ S_{4} & =\{p\in M:K_{12}=0\;\;\mathrm{and}\;\;K_{13}=0\}\\ S_{3} & =\{p\in M:K_{12}=0\;\;\mathrm{and}\;\;K_{23}=0\}\\ S_{2} & =\{p\in M:K_{13}=0\;\;\mathrm{and}\;\;K_{23}=0\}\\ S_{1} & =\{p\in M:K_{12}=0,K_{13}=0\;\;\mathrm{and}\;\;K_{23}=0\} \end{array}$$

which may be conveniently pictured as the partially ordered lattice in Figure 1. The submanifolds $S_{5}$ and $S_{6}$ are necessary for the definition of the information-geometric PID [3] and the others will be considered in the sequel. Lattices similar to that in Figure 1 appear in [8,35,36] in relation to information decomposition, and in [37] who consider dually flat manifolds on posets. See also [38], and references therein, for the use of a variety of lattices of models in statistical work.

Hierarchical chains of submanifolds were considered in [31] but here the submanifolds are not all in a hierarchical chain due to the presence of two antichains: $\left\{S_{2},S_{3},S_{4}\right\}$ and $\{S_{5},S_{6},S_{7}\}.$ There are, however, several useful chains within the lattice. Of particular relevance here are the chains $\left\{S_{2},S_{5},M\right\}$, $\left\{S_{2},S_{6},M\right\}$ and $\left\{S_{2},M\right\}$. Application of Amari’s mixed-cut coordinates [31] and calculation of divergences produces measures of mutual information that are of direct relevance in PID (as was noted by [3] for three scalar random variables) in that the equations

$$\begin{array}{cc} I[X_{1};X_{2};X_{3}] & =I\left[X_{1};X_{3}\right]+I\left[X_{2};X_{3}|X_{1}\right]\\ \; & =I\left[X_{2};X_{3}\right]+I\left[X_{1};X_{3}|X_{2}\right] \end{array}$$

are obtained—and they are standard results in information theory based on the chain rule for mutual information [39]. These are nice illustrations of Amari’s method.

We now consider *m*-projections from the pdf p∈M to each of the submanifolds, $S_{1}-S_{7}$ [31]. It is easy to find the pdf in each submanifold that is closest to the given pdf in M, *p*, in terms of Kullback–Leibler (KL) divergence [40], ([Ch. 4]). They are given in Figure 1. We know [34,40] that setting a block of the inverse covariance for a multivariate Gaussian distribution to zero expresses a conditional dependence between the variables involved. For example, consider $S_{5}$. On this submanifold $K_{23}=0$ and so $X_{2}$ and $X_{3}$ are conditionally independent given a value for $X_{1}$. Therefore, this pdf, which we denote by $p_{5}$, has the form $p_{5}\left(x_{1},x_{2},x_{3}\right)=p\left(x_{2}|x_{1}\right)p\left(x_{3}|x_{1}\right)p\left(x_{1}\right).$ On submanifold $S_{2}$, there are two conditional independences and so $X_{3}$ and the pair $\left(X_{1},X_{2}\right)$ are independent and the closest pdf in $S_{2}$ to the pdf *p* has the form $p_{2}\left(x_{1},x_{2},x_{3}\right)=p\left(x_{1},x_{2}\right)p\left(x_{3}\right).$

The probability distributions defined by these information projections could also have been obtained by the method of maximum entropy, subject to constraints on model interactions [31], and they were obtained in this manner in [22] by making use of Gaussian graphical models [34,40].

We now mention important results from information geometry which are crucial for defining a PID [3]. Consider the pdfs $p_{5},p_{6},p$ belonging to the submanifolds $S_{5},S_{6}$, and to manifold *M*, and the *e*-geodesic passing through $p_{5}$ and $p_{6}$. Then, any pdf on this *e*-geodesic path is also a zero-mean multivariate Gaussian pdf [41], ([Ch. 1]). We denote such a pdf by $p_{t}$. It has covariance matrix $\Sigma_{t}$, defined by

(3) $$\Sigma_{t}^{-1}=\left(1-t\right)\Sigma_{5}^{-1}+t\Sigma_{6}^{-1}$$

provided that $\Sigma_{t}^{-1}$ is positive definite. We consider also an *m*-geodesic from *p* to $p_{t}$. Then, by standard results [31,33], this *m*-geodesic meets the *e*-geodesic through $p_{5}$ and $p_{6}$ at a unique pdf $p_{t^{*}}$ such that generalized Pythagorean relationships hold in terms of the KL divergence:

(4) $$\begin{array}{cc} D(p||p_{5}) & =D(p||p_{t^{*}})+D(p_{t^{*}}\left|\right|p_{5}) \end{array}$$

(5) $$\begin{array}{cc} D(p||p_{6}) & =D(p||p_{t^{*}})+D(p_{t^{*}}\left|\right|p_{6}) \end{array}$$

The pdf $p(t^{*})$ minimizes the KL divergence between the pdf *p* in M and the pdf $p_{t}$ which lies on the *e*-geodesic which passes through pdfs $p_{5}$ and $p_{6}$.

### 2.3. The Partial Information Decomposition

Williams and Beer [1] introduce a framework called the partial information decomposition (PID) which decomposes the joint mutual information between a target and a set of multiple predictor variables into a series of terms reflecting information which is shared, unique or synergistically available within and between subsets of predictors. The joint mutual information, conditional mutual information and bivariate mutual information are defined as follows.

$$\begin{array}{cc} I[X_{1},X_{2};X_{3}] & =\;\int \int \int \;p\left(x_{1},x_{2},x_{3}\right)\log\frac{p(x_{1},x_{2},x_{3})}{p\left(x_{1},x_{2}\right)p\left(x_{3}\right)}\;dx_{1}dx_{2}dx_{3},\\ I[X_{1};X_{3}|X_{2}] & =\;\int \int \int \;p\left(x_{1},x_{2},x_{3}\right)\log\frac{p\left(x_{1},x_{2},x_{3}\right)p\left(x_{2}\right)}{p\left(x_{1},x_{2}\right)p\left(x_{2},x_{3}\right)}\;dx_{1}dx_{2}dx_{3},\\ I[X_{1};X_{3}] & =\;\int \int \;p\left(x_{1},x_{3}\right)\log\frac{p(x_{1},x_{3})}{p\left(x_{1}\right)p\left(x_{3}\right)}\;dx_{1}dx_{3}, \end{array}$$

Here, we focus on the case of two vector sources, $X_{1},X_{2}$, and a vector target $X_{3}$. Adapting the notation of [42], we express the joint mutual information in four terms as follows:
$\mathrm{Unq}1\equiv I_{unq}\left[X_{1};X_{3}|X_{2}\right]$denotes the unique information that $X_{1}$ conveys about $X_{3}$;$\mathrm{Unq}2\equiv I_{unq}\left[X_{2};X_{3}|X_{1}\right]$is the unique information that $X_{2}$ conveys about $X_{3}$;$\mathrm{Shd}\equiv I_{shd}\left[X_{1},X_{2};X_{3}\right]$gives the common (or redundant or shared) information that both $X_{1}$ and $X_{2}$ have about $X_{3}$;$\mathrm{Syn}\equiv I_{syn}\left[X_{1},X_{2};X_{3}\right]$is the synergy or information that the joint vector $\left(X_{1},X_{2}\right)$ has about $X_{3}$ that cannot be obtained by observing $X_{1}$ and $X_{2}$ separately.
It is possible to make deductions about a PID by using the following four equations which give a link between the components of a PID and certain classical Shannon measures of mutual information. The following are from ([42] Equations (4) and (5)), with amended notation; see also [1].

(6) $$\begin{array}{cc} I[X_{1};X_{3}] & =\mathrm{Unq}1+\mathrm{Shd}, \end{array}$$

(7) $$\begin{array}{cc} I[X_{2};X_{3}] & =\mathrm{Unq}2+\mathrm{Shd}, \end{array}$$

(8) $$\begin{array}{cc} I[X_{1};X_{3}|X_{2}] & =\mathrm{Unq}1+\mathrm{Syn}, \end{array}$$

(9) $$\begin{array}{cc} I[X_{2};X_{3}|X_{1}] & =\mathrm{Unq}2+\mathrm{Syn}. \end{array}$$

Also, the joint mutual information may be written as

(10) $$I[X_{1},X_{2};X_{3}]=\mathrm{Syn}+\mathrm{Unq}1+\mathrm{Unq}2+\mathrm{Shd}.$$

The Equations (6)–(9) are of rank 3 and so it is necessary to provide a value for any one of the components, and then the remaining terms can be easily calculated. The initial formulation of [1] was based on quantifying the shared information and deriving the other quantities, but others have focussed on quantifying unique information or synergy directly [4,5,8]. Also, the following form [16] of the interaction information [2] will be useful. It was shown [1] to be equal to the difference in Syn—Shd.

(11) $$II\left[X_{1};X_{2};X_{3}\right]=I\left[X_{1};X_{2}|X_{3}\right]-I\left[X_{1};X_{2}\right].$$

## 3. Results

### 3.1. A PID for Gaussian Vector Sources and a Gaussian Vector Target

We now apply the results from the previous two sections in order to derive a partial information decomposition by making use of the method defined in [3]. The following lemma will confirm that without any loss of generality, we may assume, for all of the multivariate normal distributions considered herein, that the mean vector can be taken to be 0 and the covariance matrix of Z, defined on $R^{m}$, where $m=n_{1}+n_{2}+n_{3}$, can have the form

(12) $$\Sigma =\left[\begin{array}{ccc} I_{n_{1}} & P & Q\\ P^{T} & I_{n_{2}} & R\\ Q^{T} & R^{T} & I_{n_{3}} \end{array}\right],$$

where the matrices P,Q,R are of size $n_{1}\times n_{2},n_{1}\times n_{3},n_{2}\times n_{3}$, respectively, and are the cross-covariance (correlation) matrices between the three pairings of the three random vectors $X_{1},X_{2},X_{3},$ and so

$$E\left(X_{1}X_{2}^{T}\right)=P,\;E\left(X_{1}X_{3}^{T}\right)=Q,\;E\left(X_{2}X_{3}^{T}\right)=R.$$

The calculation of the partial information coefficients will involve the computation of KL divergences [43] between two multivariate Gaussian distributions associated with two submanifolds in the lattice, defined in Figure 1; see Lemma 1, with proof in Appendix C. These probability distributions will have two features in common: they each have the same partitioned mean vector and also the same variance–covariance matrices for the random vectors $X_{1}$, $X_{2}$ and $X_{3}$, but different cross covariance matrices for each pair of the random vectors $X_{1}$, $X_{2}$ and $X_{3}$.

**Lemma** **1.** *Consider two multivariate Gaussian pdfs, $f_{1}$ and $f_{2}$, which have the same partitioned mean vector, $\mu ={\left[\mu_{1}^{T},\mu_{2}^{T},\mu_{3}^{T}\right]}^{T}$, and conformably partitioned m×m covariance matrices*

(13) $$\Phi =\left[\begin{array}{ccc} \Sigma_{11} & \Phi_{12} & \Phi_{13}\\ \Sigma_{12}^{T} & \Sigma_{22} & \Phi_{23}\\ \Phi_{13}^{T} & \Phi_{23}^{T} & \Sigma_{33} \end{array}\right]\;\mathrm{and}\;\Lambda =\left[\begin{array}{ccc} \Sigma_{11} & \Lambda_{12} & \Lambda_{13}\\ \Lambda_{12}^{T} & \Sigma_{22} & \Lambda_{23}\\ \Lambda_{13}^{T} & \Lambda_{23}^{T} & \Sigma_{33} \end{array}\right]$$

*respectively, where the diagonal blocks $\Sigma_{11},\Sigma_{22},and\Sigma_{33}$ are square.*

*Then, the Kullback–Liebler divergence $D(f_{1}||f_{2})$ does not depend on the mean vector **μ**, nor does it depend directly on the variance–covariance matrices $\Sigma_{11},\Sigma_{22},\Sigma_{33}$. The divergence is equal to*

(14) $$D(f_{1}\left|\right|f_{2})=\frac{1}{2}\left(\log\frac{|\Lambda_{1}|}{|\Phi_{1}|}-m+\mathrm{Tr}\left(\Phi_{1}^{-1}\Lambda_{1}\right)\right),$$

*where*

$$\Phi_{1}=\left[\begin{array}{ccc} I_{n_{1}} & P_{12} & P_{13}\\ P_{12}^{T} & I_{n_{2}} & P_{23}\\ P_{13}^{T} & P_{23}^{T} & I_{n_{3}} \end{array}\right]\;\mathrm{and}\;\Lambda_{1}=\left[\begin{array}{ccc} I_{n_{1}} & L_{12} & L_{13}\\ L_{12}^{T} & I_{n_{2}} & L_{23}\\ L_{13}^{T} & L_{23}^{T} & I_{n_{3}} \end{array}\right]$$

*with*

$$P_{ij}=\Sigma_{ii}^{-\frac{1}{2}}\Phi_{ij}\Sigma_{jj}^{-\frac{1}{2}},\;L_{ij}=\Sigma_{ii}^{-\frac{1}{2}}\Lambda_{ij}\Sigma_{jj}^{-\frac{1}{2}},\;\left(i,j=1,2,3\;;i\leq j\right),$$

*which are the respective cross-correlation matrices among $X_{1},X_{2},X_{3}$. The KL divergence depends only on these cross-correlation matrices.*

### 3.2. Covariance Matrices

Table 1 gives the covariance matrices corresponding to each of the projected distributions $p_{1}-p_{7}$ on the submanifolds. It is known from Gaussian graphical models [34,40] that the probability distributions associated with submanifolds $S_{5}$ and $S_{6}$ are defined by setting $K_{23}=0$ and $K_{13}=0$, respectively, in the precision matrix *K*. These conditions were shown in [22] to be equivalent to the equations $R=P^{T}Q$ and Q=PR, respectively. From Table 1, we see that the covariance matrices for pdfs $p_{5}$ and $p_{6}$ have the following form.

(15) $$\Sigma_{5}=\left[\begin{array}{ccc} I_{n_{1}} & P & Q\\ P^{T} & I_{n_{2}} & P^{T}Q\\ Q^{T} & Q^{P} & I_{n_{3}} \end{array}\right],\;\mathrm{and}\;\Sigma_{6}=\left[\begin{array}{ccc} I_{n_{1}} & P & PR\\ P^{T} & I_{n_{2}} & R\\ R^{T}P^{T} & R^{T} & I_{n_{3}} \end{array}\right].$$

The following lemma, which is proved in Appendix D, gives some useful results on determinants that will be used in the sequel.

**Lemma** **2.** *The determinants of the matrices $\Sigma_{5},\Sigma_{6}$ are given by*

$$|\Sigma_{5}\left|=\right|I_{n_{2}}-P^{T}P||I_{n_{3}}-Q^{T}Q|\;\mathrm{and}\;|\Sigma_{6}\left|=\right|I_{n_{2}}-P^{T}P||I_{n_{3}}-R^{T}R|.$$

*Also,*

$$\Sigma_{5}=\Sigma_{6}\Leftrightarrow Q=0\;\;\mathrm{and}\;\;R=0\Leftrightarrow I\left(X_{1},X_{2};X_{3}\right)=0.$$

### 3.3. Feasible Values for the Parameter t

From (3), the *m*-projection from manifold *M* to the *e*-geodesic passing through the pdfs $p_{5}$ and $p_{6}$ meets in general at pdf $p_{t}$ which has covariance matrix $\Sigma_{t}$ defined by

$$\Sigma_{t}^{-1}=\left(1-t\right)\Sigma_{5}^{-1}+t\;\Sigma_{6}^{-1},$$

and $\Sigma_{t}$ must be positive definite. Therefore, when finding the optimal pdf $p_{t^{*}}$, we require to constrain the values of the parameter *t* to be such that $\Sigma_{t}$ is positive definite. We define the set of feasible values for *t* as

(16) $$F=\{t\in R:\Sigma_{t}\;\;\mathrm{is}\;\mathrm{positive}\;\mathrm{definite}\}.$$

*F* is a closed interval in R of the form [−a,1+b], where a,b>0. The interior of *F*—the open interval [−a,1+b]—is an open convex set. To enable the derivation of explicit results, it is useful to define the matrix $\Sigma_{t}^{\prime }$ by

(17) $$\Sigma_{t}^{\prime \;-1}=\left(1-t\right)\Sigma_{6}+t\Sigma_{5}.$$

We also require a feasible value $t^{*}$ for *t* when working with the matrix $\Sigma_{t}^{\prime }$, and so we define the set *G* of feasible values as follows

(18) $$G=\{t\in R:\Sigma_{t}^{\prime }\;\;\mathrm{is}\;\mathrm{positive}\;\mathrm{definite}\}.$$

It turns out that the sets of feasible values F,G are actually the same set, as stated in the following lemma, which is proved in Appendix E, and this fact allows us to infer that $\Sigma_{t}^{\prime }$ is positive definite when $\Sigma_{t}$ is.

**Lemma** **3.** *If the parameter t belongs to the closed interval [0,1], then the matrices $\Sigma_{t}$ and $\Sigma_{t}^{\prime }$ are both positive definite. Also, the two feasible sets F and G defined above in (16) and (18) are equal.*

### 3.4. A Convex Optimisation Problem

The optimal value $t^{*}$ of the parameter *t* is defined by

(19) $$t^{*}=\underset{t\in F}{\mathrm{arg min}}\;\;D(p||p_{t})$$

The following lemma, with proof in Appendix F, provides details of the optimisation required to find $t^{*}$.

**Lemma** **4.** *For $t\in F^{\circ }$, we define the real valued function g by $g\left(t\right)=D(p||p_{t})$. Then,*

(20) $$\mathrm{Tr}(\Sigma_{t}^{-1}\Sigma )=m,$$

*and*

(21) $$g\left(t\right)=\frac{1}{2}\log\frac{|I_{n_{2}}-P^{T}{P|}^{2}|I_{n_{3}}-Q^{T}Q||I_{n_{3}}-R^{T}R|}{\left|\Sigma \right|}-\frac{1}{2}\log\left|\left(1-t\right)\Sigma_{6}+t\Sigma_{5}\right|$$

*Provided that the joint mutual information is positive, the minimization of g subject to the constraint $t\in F^{\circ }$, an open convex set, is a strictly convex problem, and the optimal value $t^{*}$ is unique.*

*The minimum value of g is equal to*

(22) $$\frac{1}{2}\log\frac{\left|\begin{array}{c} I_{n_{2}}-P^{T}P \end{array}\right|\left|\begin{array}{c} I_{n_{3}}-Q^{T}Q \end{array}\right|\left|\begin{array}{c} I_{n_{3}}-R^{T}R \end{array}\right|}{\left|\Sigma \right|}+\frac{1}{2}\log\frac{1}{d(t^{*})},$$

*where the determinant d(t) is defined by*

(23) $$d\left(t\right)=\left|\begin{array}{c} I_{n_{3}}-{\left(1-t\right)}^{2}Q^{T}Q-t\left(1-t\right)\left(R^{T}P^{T}Q+Q^{T}PR\right)-t^{2}R^{T}R \end{array}\right|,\;\left(t\in F\right)$$

*Alternatively, the minimum could occur at either endpoint of F.*

We now define the PID components.

### 3.5. Definition of the PID Components

Following the proposal in [3], we define the synergy of the system to be

(24) $$\mathrm{Syn}=D(p||p_{t}^{*})$$

and by Lemma 1 and (20) the expression for the synergy is

(25) $$\mathrm{Syn}=\frac{1}{2}\log\frac{|\Sigma_{t^{*}}|}{\left|\Sigma \right|}-\frac{1}{2}m+\frac{1}{2}\mathrm{Tr}\left(\Sigma_{t^{*}}^{-1}\Sigma \right)=\frac{1}{2}\log\frac{|\Sigma_{t^{*}}|}{\left|\Sigma \right|}.$$

Before defining the other PID terms, we require the following lemma, with proof in Appendix G.

**Lemma** **5.** *The trace terms required in the definitions of the unique information are both equal to m:*

(26) $$\mathrm{Tr}\left(\Sigma_{5}^{-1}\Sigma_{t^{*}}\right)=m\;\mathrm{and}\;\mathrm{Tr}\left(\Sigma_{6}^{-1}\Sigma_{t^{*}}\right)=m.$$

From (4), we know that

(27) $$D(p||p_{5})=D(p||p_{t}^{*})+D(p_{t}^{*}\left|\right|p_{5})$$

and we define the unique information in the system that is due to source $X_{2}$ to be

(28) $$\mathrm{Unq}2=D(p_{t}^{*}||p_{5})$$

as in [3]. By (5), we also have that

(29) $$D(p||p_{6})=D(p||p_{t}^{*})+D(p_{t}^{*}\left|\right|p_{6})$$

and we define the unique information in the system that is due to source $X_{1}$ to be

(30) $$\mathrm{Unq}1=D(p_{t}^{*}||p_{6}).$$

as in [3]. Finding the optimal point, $t^{*}$, of minimisation of the KL divergence $D(p||p_{t})$, and the orthogonality provided by the generalised Pythagorean theorems, define a clear connection between the geometry of the tangent space to manifold *M* and the definition of the information-geometric PID developed herein.

By using two of the defining equations of a PID (6) and (7), there are two possible expressions for the shared information, Shd, in the system:

(31) $$I\left(X_{1};X_{3}\right)-\mathrm{Unq}1\;\mathrm{or}\;I\left(X_{2};X_{3}\right)-\mathrm{Unq}2.$$

Using the result in Lemma 1, we may write the unique information terms as follows. The unique information provided by $X_{2}$ is defined to be

$$\mathrm{Unq}2=D(p_{t^{*}}\left|\right|p_{5})=\frac{1}{2}\log\frac{|\Sigma_{5}|}{|\Sigma_{t^{*}}|}-\frac{1}{2}m+\frac{1}{2}\mathrm{Tr}\left(\Sigma_{5}^{-1}\Sigma_{t^{*}}\right)=\frac{1}{2}\log\frac{|\Sigma_{5}|}{|\Sigma_{t^{*}}|}.$$

by Lemma 5.

The unique information provided by $X_{1}$ is defined to be

$$\mathrm{Unq}1=D(p_{t^{*}}\left|\right|p_{6})=\frac{1}{2}\log\frac{|\Sigma_{6}|}{|\Sigma_{t^{*}}|}-\frac{1}{2}m+\frac{1}{2}\mathrm{Tr}\left(\Sigma_{6}^{-1}\Sigma_{t^{*}}\right)=\frac{1}{2}\log\frac{|\Sigma_{6}|}{|\Sigma_{t^{*}}|}.$$

by Lemma 5.

### 3.6. The $I_{\mathrm{ig}}$ PID

Explicit expressions for the PID components are given in Proposition 1, with proof in Appendix H.

**Proposition** **1.** *The partial information decomposition $I_{ig}$ for the zero-mean multivariate Gaussian system defined in *(12)* has the following components.*

$$\begin{array}{cc} \mathrm{Syn} & =\frac{1}{2}\log\frac{\left|\begin{array}{c} I_{n_{2}}-P^{T}P \end{array}\right|\left|\begin{array}{c} I_{n_{3}}-Q^{T}Q \end{array}\right|\left|\begin{array}{c} I_{n_{3}}-R^{T}R \end{array}\right|}{\left|\Sigma \right|}+\frac{1}{2}\log\frac{1}{d(t^{*})}\\ \mathrm{Unq}1 & =\frac{1}{2}\log\frac{1}{\left|\begin{array}{c} I_{n_{3}}-Q^{T}Q \end{array}\right|}-\frac{1}{2}\log\frac{1}{d(t^{*})}\\ \mathrm{Unq}2 & =\frac{1}{2}\log\frac{1}{\left|\begin{array}{c} I_{n_{3}}-R^{T}R \end{array}\right|}-\frac{1}{2}\log\frac{1}{d(t^{*})}\\ \mathrm{Shd} & =\frac{1}{2}\log\frac{1}{d(t^{*})}. \end{array}$$

*where the determinant d(t) is defined by*

(32) $$d\left(t\right)=\left|\begin{array}{c} I_{n_{3}}-{\left(1-t\right)}^{2}Q^{T}Q-t\left(1-t\right)\left(R^{T}P^{T}Q+Q^{T}PR\right)-t^{2}R^{T}R \end{array}\right|,\;\left(t\in F\right)$$

*and F is the interval of real values of t for which $\Sigma_{t}$ is positive definite.*

*The two possible expressions for the shared information in *(31)* are equal.*

Theoretical properties of the $I_{\mathrm{ig}}$ PID are presented in Proposition 2, with proof in Appendix I.2.

**Proposition** **2.** *The PID defined in Proposition 1 possesses the Williams–Beer properties of non-negativity, self-redundancy, symmetry and monotonicity.*

### 3.7. Some Examples and Illustrations

**Example** **1.** *Prediction of calcium contents.*

This dataset was considered in [22]. The $I_{\mathrm{ig}}$ PID developed here, along with the $I_{\mathrm{dep}}$ PID [22] and $I_{\mathrm{mmi}}$ PID [20], was applied using data on 73 women involving one set of predictors $X_{1}$ (Age, Weight, Height), another set of two predictors $X_{2}$ (diameter of os calcis, diameter of radius and ulna), and target $X_{3}$ (calcium content of heel and forearm). The following results were obtained.
**PID**$t^{*}$**Unq1****Unq2****Shd****Syn**$I_{\mathrm{ig}}$ 0.24080.35810.03040.07280.1904$I_{\mathrm{dep}}$ 
0.40770.08000.02320.1408$I_{\mathrm{mmi}}$ 
0.327700.10320.2209

**Figure 2:**
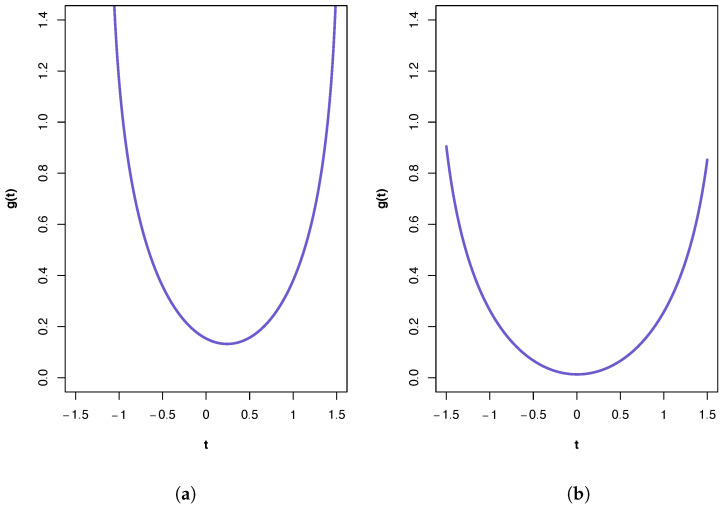
Plots of the ‘synergy’ function g(t) for the two calcium datasets. (**a**) First calcium dataset. The feasible range for *t* is (−1.13, 1.56), with $t^{*}=0.2408$. (**b**) Second calcium dataset. The feasible range for *t* is (−1.67, 1.69), with $t^{*}=0.0027$.

A plot of the ‘synergy’ function g(t) is shown in Figure 2a. All three PIDs indicate the presence of synergy and a large component of unique information due to the variables in $X_{1}$. The $I_{\mathrm{ig}}$ PID suggests the transmission of more of the joint mutual information as shared and synergistic information and correspondingly less unique information due to either source vector than does the $I_{\mathrm{dep}}$ PID. This is true also for the results from the $I_{\mathrm{mmi}}$ PID, but it has higher values for synergistic and shared information and a lower value for Unq1 than those produced by the $I_{\mathrm{ig}}$ PID. It was shown in [22] that pdf *p* in manifold M provides a better fit to these data than any of the submanifold distributions. This pdf contains pairwise cross-correlation between the vectors $X_{1}$ and $X_{3}$, and between $X_{2}$ and $X_{3}$. Hence, it is no surprise to find that a relatively large Unq1 component. One might also anticipate a large value for Unq2. That this is not the case is explained, at least partly, by the presence of unique information asymmetry, in that the mutual information between $X_{1}$ and $X_{3}$ (0.4309) is much larger than that between $X_{2}$ and $X_{3}$ (0.1032) and also bearing in mind the constraints imposed by (6)–(10).

The PIDs were also computed with the same $X_{1}$ and $X_{3}$ but taking $X_{2}$ to be another set of four predictors (surface area, strength of forearm, strength of leg, area of os calcis). The following results were obtained.
**PID**$t^{*}$**Unq1****Unq2****Shd****Syn**$I_{\mathrm{ig}}$0.00270.35220.00000.07870.0186$I_{\mathrm{dep}}$ 
0.37080.01860.06010$I_{\mathrm{mmi}}$ 
0.352200.07870.0186

A plot of the ‘synergy’ function g(t) is shown in Figure 2b. In this case, the PIDs obtained from all three methods are very similar, with the main component being unique information due to the variables in $X_{1}$. The PIDs indicate almost zero synergy and almost zero unique information due to the variables in $X_{2}$. In [22], it was shown that the best of the pdfs is $p_{5}$ associated with submanifold $S_{5}$. If this model were to hold exactly, then a PID must have Syn and Unq2 components that are equal to zero. Therefore, all three PIDs perform very well here, and the fact that the Unq1 component is much larger than the Shd component is due to unique information asymmetry, since the mutual information between $X_{2}$ and $X_{3}$ is only 0.0787. In this dataset, the $I_{\mathrm{ig}}$ PID suggests the transmission just a little more of the joint mutual information as shared and synergistic information and correspondingly less unique information due to either source vector than does the $I_{\mathrm{dep}}$ PID. The $I_{\mathrm{ig}}$ and $I_{\mathrm{mmi}}$ PIDs produce identical results (to 4 d.p.).

When working with real or simulated data, it is important to use the correct covariance matrix. In order to use the results given in Proposition 1, it is essential that the input covariance matrix has the structure of Σ, as given in (12). Further detail is provided in Appendix J.

**Example** **2.** *PID expectations and exact results.*

Since there is no way to know the true PID for any given dataset it is useful to consider situations under which some values of the PID components can be predicted, and this approach has been used in developments of the topic. Here, we consider such expectations provided by the pdfs associated with the submanifolds $S_{3}-S_{7}$, defined in Figure 1. In submanifold $S_{3}$, the source $X_{2}$ is independent of both the other source $X_{1}$ and the target $X_{3}$. Hence, we expect only unique information due to source $X_{1}$ to be transmitted. Submanifold $S_{4}$ is similar but we expect only unique information due to source $X_{2}$ to be transmitted. In manifold $S_{5}$, $X_{2}$ and $X_{3}$ are conditionally independent given a value for $X_{1}$. Hence, from (9), we expect the Unq2 and Syn components to be zero. Similarly, for $S_{6}$, we expect the Unq1 and Syn components to be equal to zero, by (8). On submanifold $S_{7}$, the sources $X_{1},X_{2}$ are conditionally independent given a value for the target $X_{3}$ (which does not mean that the sources are marginally independent). Since the target $X_{3}$ interacts with both source vectors, one might expect some shared information as well as unique information from both sources, and also perhaps some synergy. Here, from (11), the interaction information must be negative or zero, and so we can expect to see transmission of more shared information than synergy.

We will examine these expectations by using the following multivariate Gaussian distribution (which was used in [22]). The matrices P,Q,R are given an equi-cross-correlation structure in which all the entries are equal within each matrix:

(33) $$P=p1_{n_{1}}1_{n_{2}}^{T},\;Q=q1_{n_{1}}1_{n_{3}}^{T},\;R=r1_{n_{2}}1_{n_{3}}^{T},$$

where p,q,r denote here the constant cross correlations within each matrix and $1_{n}$ denotes an n-dimensional vector whose entries are each equal to unity.

The values of (p,q,r) are taken to be (−0.15,0.15,0.15), with $n_{1}=3$, $n_{2}=4,n_{3}=3$. Covariance matrices for pdfs $p_{3}-p_{7}$ were computed using the results in Table 1. Thus, we have the exact covariance matrices which can be fed into the $I_{\mathrm{ig}}$, $I_{\mathrm{dep}}$ and $I_{\mathrm{mmi}}$ algorithms. The PID results are displayed in Table 2.

From Table 2, we see that all three PIDs meet the expectations exactly for pdfs $p_{3}-p_{6}$, with only unique information transmitted when the pdfs $p_{3},p_{4}$, are true, respectively, and zero unique for the relevant component and zero synergy when the models $p_{5},p_{6}$ are true, respectively. When model $p_{8}$ is the true model, we find that the $I_{\mathrm{ig}}$ and $I_{\mathrm{dep}}$ PIDs produce virtually identical results: the joint mutual information is transmitted almost entirely as synergistic information. The $I_{\mathrm{mmi}}$ PID is slightly different, with less unique information transmitted about the variables in $X_{2}$, and more shared and synergistic information transmitted than with the other two PIDs. The PIDs produce very different results for pdf $p_{7}$, although, as expected, they do express more shared information than synergy. When this model is satisfied, $I_{\mathrm{dep}}$ sets the synergy to 0, even if there is no compelling reason to support this. This curiosity is mentioned and illustrated in [22]. On the other hand, the $I_{\mathrm{ig}}$ PID suggests that each of the four components contributes to the transmission of the joint mutual information, with unique information due to $X_{2}$ and shared information making more of a contribution than the other two components. The $I_{\mathrm{mmi}}$ PID transmits a higher percentage of the joint information as shared and synergistic information, and a smaller percentage due to the variables in $X_{2}$, than is found with $I_{\mathrm{ig}}$; these differences are much stronger when comparison is made with the corresponding $I_{\mathrm{dep}}$ components. As with model *p*, it appears that the setting of the Unq1 component in $I_{\mathrm{ig}}$ to zero has been translated into its percentage being subtracted from the Unq2 component and added to both the Shd and Syn components in $I_{\mathrm{ig}}$ to produce $I_{\mathrm{mmi}}$.

**Example** **3.** *Some simulations.*

Taking the same values of p,q,r and $n_{1},n_{2},n_{3}$ as in the previous example, a small simulation study was conducted. From each of the pdfs, $p_{3}-p_{7},p$, a simple random sample of size 1000 was generated from the 10-dimensional distribution, a covariance matrix estimated from the data and the $I_{\mathrm{ig}}$, $I_{\mathrm{dep}}$ and $I_{\mathrm{mmi}}$ algorithms were applied. This procedure was repeated 1000 times. In order to make the PID results from the sample of 1000 datasets comparable each PID was normalized by dividing each of its components by the joint mutual information; see (10). A summary of the results is provided in Table 3. We focus here on the comparison of $I_{\mathrm{ig}}$ and $I_{\mathrm{dep}}$, and also $I_{\mathrm{ig}}$ and $I_{\mathrm{mmi}}$, since $I_{\mathrm{dep}}$ has been compared with $I_{\mathrm{mmi}}$ for Gaussian systems [22].

$I_{\mathrm{ig}}$ *vs.* $I_{\mathrm{dep}}$

For pdf *p*, the $I_{\mathrm{ig}}$ and $I_{\mathrm{dep}}$ PIDs produce very similar results in terms of both median and range, and the median results are very close indeed to the corresponding exact values in Table 2. For pdf $p_{7}$, the differences between the PID components found in Table 2 persist here although each PID, respectively, produces median values of their components that are close to the exact results in Table 2. For the other four pdfs, there are some small but interesting differences among the results produced by the two PID methods. The $I_{\mathrm{ig}}$ method has higher median values for synergy and shared information than for the unique information, when compared against the corresponding exact values in Table 2. In particular, the values of unique information given by $I_{\mathrm{ig}}$ are much lower than expected for pdfs $p_{3},p_{4},p_{6}$, and the levels of synergy are larger than expected particularly for pdfs $p_{3}$ and $p_{5}$. On the other hand, the $I_{\mathrm{dep}}$ PID tends to have larger values for the unique information, and lower values for synergy, especially for datasets generated from pdfs $p_{3}$, $p_{4}$ and $p_{5}$. For models $p_{3}-p_{6}$, $I_{\mathrm{dep}}$ has median values of synergy that are closer to the corresponding exact values than those produced by $I_{\mathrm{ig}}$. The suggestion that the $I_{\mathrm{ig}}$ method can produce more synergy and shared information than the $I_{\mathrm{dep}}$ method, given the same dataset, is supported by the fact that for all the pdfs and all 6000 datasets considered, the $I_{\mathrm{ig}}$ method produced greater levels of synergy and shared information and smaller values of the unique information in *every* dataset. This raises a question of whether such a finding is generally the case and whether there is this type of a systematic difference between the methods. In the case of scalar variables, it is easy to derive general analytic formulae for the $I_{\mathrm{ig}}$ PID components and such a systematic difference is present in this case.

$I_{\mathrm{ig}}$ *vs.* $I_{\mathrm{mmi}}$

The $I_{\mathrm{ig}}$ and $I_{\mathrm{mmi}}$ PIDs produce similar results for the datasets generated from pdf *p*, although the $I_{\mathrm{mmi}}$ PID suggests the transmission of more shared and synergistic information and less unique information than does $I_{\mathrm{ig}}$. For pdf $p_{7}$, the differences between the PID results are much more dramatic, with the $I_{\mathrm{mmi}}$ PID allocating an additional 15% of the joint mutual information to be shared and the synergistic information, and correspondingly 15% less of the unique information. Both methods produce almost identical summary statistics on the datasets generated from pdfs $p_{3}-p_{6}$. Since the same patterns are present for all four distributions, we discuss the results for pdf $p_{5}$ as an exemplar and compare them with the corresponding exact values in Table 2. The results for component Unq1 show that both methods produce an underestimate of approximately 7%, on average, of the joint mutual information. The median values of Unq2 are close to those expected. The underestimates on the Unq1 component are coupled with overestimates, on average, for the shared and synergistic components; they are 2.6% and 4.3%, respectively, with the $I_{\mathrm{ig}}$ method, and 3.1% and 4.7%, respectively, with $I_{\mathrm{mmi}}$.

As to be expected with percentage data, the variation in results for each component tends to be larger for values that are not extreme and much smaller for the extreme values. Also, the optimal values of $t^{*}$ are shown in Table 3. They were all found to be in the range [0, 1], except for 202 of the datasets generated from pdf $p_{5}$ or $p_{6}$.

## 4. Discussion

For the case of multivariate Gaussian systems with two vector inputs and a vector output, results have been derived using standard theorems from information geometry in order to develop simple, almost exact formulae for the $I_{\mathrm{ig}}$ PID, thus extending the scope of the work of [3] on scalar inputs and output. The formulae require one parameter to be determined by a simple, constrained convex optimisation. In addition, it has been proved that this $I_{\mathrm{ig}}$ PID algorithm satisfies the desirable theoretical properties of non-negativity, self-redundancy, symmetry and monotonicity, first postulated by Williams and Beer [1]. These results strengthen the confidence that one might have in using the $I_{\mathrm{ig}}$ method to separate the joint mutual information in a multivariate Gaussian system into shared, unique and synergistic components. The examples demonstrate that the $I_{\mathrm{ig}}$ method is simple to use and a small simulation study reveals that it is fairly robust, although in some of the scenarios considered the $I_{\mathrm{ig}}$ method produced more synergy and shared information than expected, and correspondingly less unique information; in some other scenarios, it performed as expected. Comparison of the $I_{\mathrm{ig}}$ and $I_{\mathrm{dep}}$ algorithms reveal that they can produce exactly the same, or very similar, results in some scenarios, but in other situations, it is clear that the $I_{\mathrm{ig}}$ method tends to have larger levels of shared information and synergy, and correspondingly, lower levels of unique information when compared with the results from the $I_{\mathrm{dep}}$ method.

For datasets generated from pdfs $p_{3}-p_{6}$, the PIDs produced using the $I_{\mathrm{ig}}$ and $I_{\mathrm{mmi}}$ methods are, on average, very similar indeed, and both methods overestimate synergy and shared information and underestimate unique information. The extent of these biases, as a percentage of the joint mutual information, is fairly small, on average, when pdf $p_{4}$ or $p_{6}$ is the true pdf, but larger, on average, when $p_{3}$ or $p_{5}$ is the true pdf. When pdf $p_{7}$ or *p* is the true pdf, the $I_{\mathrm{mmi}}$ algorithm produces even more shared and synergistic information than obtained with the $I_{\mathrm{ig}}$ method. This effect is particularly dramatic in the case of $p_{7}$, where on average with $I_{\mathrm{mmi}}$ 82% of the joint mutual information is transmitted as shared or synergistic information, as compared with 51.5% for $I_{\mathrm{ig}}$. It appears that the fact that the $I_{\mathrm{mmi}}$ method forces one of the unique informations to be zero leads to an underestimation of the other unique information and an overestimate of both the shared and synergistic information, especially when $p_{7}$ or *p* is the true pdf and both unique information are expected to be non-zero.

While some numerical support is presented here for the hypothesis that there might be a systematic difference in this type between the $I_{\mathrm{ig}}$ and $I_{\mathrm{dep}}$ methods further research would be required to investigate this possibility. Also, the $I_{\mathrm{ig}}$ developed here is a bivariate PID and it would be of interest to explore whether the method could be extended to deal with more than two source vectors.

## Figures and Tables

**Table 1:** Submanifold probability distributions with corresponding covariance matrices (modified from [22]).

Submanifold	$\Sigma_{i}$	Submanifold	$\Sigma_{i}$
$S_{1}:p\left(x_{1}\right)p\left(x_{2}\right)p\left(x_{3}\right)$	$\left[\begin{array}{ccc} I_{n_{1}} & 0 & 0\\ 0 & I_{n_{2}} & 0\\ 0 & 0 & I_{n_{3}} \end{array}\right]$	$S_{5}:p\left(x_{2}|x_{1}\right)p\left(x_{3}|x_{1}\right)p\left(x_{1}\right)$	$\left[\begin{array}{ccc} I_{n_{1}} & P & Q\\ P^{T} & I_{n_{2}} & P^{T}Q\\ Q^{T} & Q^{T}P & I_{n_{3}} \end{array}\right]$
$S_{2}:p\left(x_{1},x_{2}\right)p\left(x_{3}\right)$	$\left[\begin{array}{ccc} I_{n_{1}} & P & 0\\ P^{T} & I_{n_{2}} & 0\\ 0 & 0 & I_{n_{3}} \end{array}\right]$	$S_{6}:p\left(x_{1}|x_{2}\right)p\left(x_{3}|x_{2}\right)p\left(x_{2}\right)$	$\left[\begin{array}{ccc} I_{n_{1}} & P & PR\\ P^{T} & I_{n_{2}} & R\\ R^{T}P^{T} & R^{T} & I_{n_{3}} \end{array}\right]$
$S_{3}:p\left(x_{1},x_{3}\right)p\left(x_{2}\right)$	$\left[\begin{array}{ccc} I_{n_{1}} & 0 & Q\\ 0 & I_{n_{2}} & 0\\ Q^{T} & 0 & I_{n_{3}} \end{array}\right]$	$S_{7}:p\left(x_{1}|x_{3}\right)p\left(x_{2}|x_{3}\right)p\left(x_{3}\right)$	$\left[\begin{array}{ccc} I_{n_{1}} & QR^{T} & Q\\ RQ^{T} & I_{n_{2}} & R\\ Q^{T} & R^{T} & I_{n_{3}} \end{array}\right]$
$S_{4}:$ $p\left(x_{2},x_{3}\right)p\left(x_{1}\right)$	$\left[\begin{array}{ccc} I_{n_{1}} & 0 & 0\\ 0 & I_{n_{2}} & R\\ 0 & R^{T} & I_{n_{3}} \end{array}\right]$	$M:p(x_{1},x_{2},x_{3})$	$\left[\begin{array}{ccc} I_{n_{1}} & P & Q\\ P^{T} & I_{n_{2}} & R\\ Q^{T} & R^{T} & I_{n_{3}} \end{array}\right]$

**Table 2:** PID results for exact pdfs, reported as a percentage of the joint mutual information.

pdf	PID	Unq1	Unq2	Shd	Syn
*p*	$I_{\mathrm{ig}}$	4.3	6.6	1.5	87.7
	$I_{\mathrm{dep}}$	4.3	6.6	1.4	87.6
	$I_{\mathrm{mmi}}$	0.0	2.3	5.8	91.9
$p_{7}$	$I_{\mathrm{ig}}$	15.0	33.2	31.7	20.1
	$I_{\mathrm{dep}}$	35.1	53.3	11.6	0.0
	$I_{\mathrm{mmi}}$	0.0	18.2	46.7	35.1
$p_{6}$	$I_{\mathrm{ig}}$	0.0	75.9	24.1	0.0
	$I_{\mathrm{dep}}$	0.0	75.9	24.1	0.0
	$I_{\mathrm{mmi}}$	0.0	75.9	24.1	0.0
$p_{5}$	$I_{\mathrm{ig}}$	75.2	0.0	24.8	0.0
	$I_{\mathrm{dep}}$	75.2	0.0	24.8	0.0
	$I_{\mathrm{mmi}}$	75.2	0.0	24.8	0.0
$p_{4}$	$I_{\mathrm{ig}}$	0.0	100.0	0.0	0.0
	$I_{\mathrm{dep}}$	0.0	100.0	0.0	0.0
	$I_{\mathrm{mmi}}$	0.0	100.0	0.0	0.0
$p_{3}$	$I_{\mathrm{ig}}$	100.0	0.0	0.0	0.0
	$I_{\mathrm{dep}}$	100.0	0.0	0.0	0.0
	$I_{\mathrm{mmi}}$	100.0	0.0	0.0	0.0

**Table 3:** PID results for simulated datasets from the pdfs, $p_{3}-p_{7},p$, reported as median (in bold) and range of the sample of percentages of the joint mutual information, apart from $t^{*}$ which gives the median and range of the actual values obtained using the $I_{\mathrm{ig}}$ algorithm.

pdf	PID	$t^{*}$	Unq1	Unq2	Shd	Syn
*p*	$I_{\mathrm{ig}}$	0.55	**4.4**	**6.7**	**1.6**	**87.3**
		(0.44, 0.63)	(2.6, 6.6)	(3.7, 9.0)	(1.1, 2.1)	(85.4, 89.2)
	$I_{\mathrm{dep}}$		**4.5**	**6.8**	**1.4**	**87.2**
			(2.8, 6.8)	(3.9, 9.3)	(1.0, 1.9)	(85.3, 89.0)
	$I_{\mathrm{mmi}}$		**0.0**	**2.3**	**6.0**	**91.7**
			(0.0, 2.9)	(3.2, 6.2)	(3.9, 8.0)	(89.2, 93.6)
$p_{7}$	$I_{\mathrm{ig}}$	0.59	**15.1**	**33.0**	**31.3**	**20.2**
		(0.47, 0.71)	(7.6, 26.5)	(19.9, 48.0)	(23.4, 37.3)	(15.2, 24.9)
	$I_{\mathrm{dep}}$		**34.3**	**52.3**	**11.8**	**0.2**
			(23.6, 46.4)	(38.9, 64.5)	(7.8, 20.3)	(0.0, 8.6)
	$I_{\mathrm{mmi}}$		**0.0**	**18.0**	**46.4**	**35.6**
			(0.0, 11.4)	(0.0, 45.9)	(30.4, 57.7)	(21.3, 45.9)
$p_{6}$	$I_{\mathrm{ig}}$	0.97	**0.1**	**72.5**	**25.0**	**2.4**
		(0.82, 1.06)	(0.0, 2.4)	(55.6, 84.7)	(11.4, 38.4)	(0.2, 8.1)
	$I_{\mathrm{dep}}$		**2.5**	**74.9**	**22.6**	**0.0**
			(0.2, 10.4)	(60.4, 88.6)	(7.6, 35.3)	(0.0, 0.0)
	$I_{\mathrm{mmi}}$		**0.0**	**72.3**	**25.1**	**2.6**
			(0.0, 0.0)	(45.7, 83.6)	(13.5, 45.4)	(0.3, 8.9)
$p_{5}$	$I_{\mathrm{ig}}$	0.06	**68.0**	**0.3**	**27.4**	**4.3**
		(−0.06, 0.20)	(50.0, 81.7)	(0.0, 2.9)	(12.5, 43.3)	(0.9, 12.1)
	$I_{\mathrm{dep}}$		**72.3**	**4.7**	**22.8**	**0.0**
			(54.9, 87.3)	(0.9, 13.3)	(6.8, 38.4)	(0.0, 0.0)
	$I_{\mathrm{mmi}}$		**67.5**	**0.0**	**27.9**	**4.7**
			(45.7, 82.6)	(0.0, 0.0)	(13.9, 45.5)	(0.9, 14.2)
$p_{4}$	$I_{\mathrm{ig}}$	0.97	**0.06**	**95.0**	**2.5**	**2.4**
		(0.91, 1.0)	(0.0, 0.7)	(83.0, 99.5)	(1.9, 7.8)	(0.3, 8.3)
	$I_{\mathrm{dep}}$		**2.2**	**97.2**	**0.3**	**0.2**
			(0.2, 8.4)	(90.7, 99.7)	(0.0, 2.6)	(0.0, 2.7)
	$I_{\mathrm{mmi}}$		**0.0**	**94.9**	**2.5**	**2.6**
			(0.0, 0.0)	(83.1, 99.3)	(0.1, 9.4)	(0.4, 8.6)
$p_{3}$	$I_{\mathrm{ig}}$	0.05	**90.9**	**0.2**	**4.5**	**4.5**
		(0.01, 0.14)	(75.7, 98.4)	(0.0, 1.8)	(0.7, 11.5)	(0.9, 11.5)
	$I_{\mathrm{dep}}$		**94.8**	**4.2**	**0.3**	**0.3**
			(86.2, 99.1)	(0.7, 12.6)	(0.0, 3.1)	(0.0, 3.5)
	$I_{\mathrm{mmi}}$		**90.6**	**0.0**	**4.6**	**4.8**
			(76.3, 97.6)	(0.0, 0.0)	(1.0, 11.9)	(1.2, 13.0)

## Data Availability

The data used in Example 1 is available from https://github.com/JWKay/PID (accessed on 17 June 2024).

## Abbreviations

The following abbreviations are used in this manuscript:

d.p.	decimal places
$I_{\mathrm{dep}}$	Idep PID for Gaussian systems
$I_{\mathrm{ig}}$	Information-geometric PID
$I_{\mathrm{mmi}}$	Immi for Gaussian systems
KL	Kullback–Leibler
pdf	probability density function
PID	Partial information decomposition

## Appendix A. Two Matrix Lemmas from [22]

**Lemma** **A1.**
*Suppose that a symmetric matrix M is partitioned as*

$$M=\left[\begin{array}{cc} A & B\\ B^{T} & C \end{array}\right],$$

*where A and C are symmetric and square. Then,*

(i) *The matrix M is positive definite if and only if A and $C-B^{T}A^{-1}B$ are positive definite.*
(ii) *The matrix M is positive definite if and only if C and $A-BC^{-1}B^{T}$ are positive definite.*
(iii) $\left|M\right|=|A||C-B^{T}A^{-1}B|.$

**Lemma** **A2.** *When the covariance matrix* Σ *in *(12)* is positive definite then the following matrices are also positive definite, and hence nonsingular:*

$$I_{n_{2}}-P^{T}P,\;\;I_{n_{1}}-PP^{T},\;\;I_{n_{3}}-R^{T}R,\;\;I_{n_{2}}-RR^{T},\;\;I_{n_{3}}-Q^{T}Q,\;\;I_{n_{1}}-QQ^{T}.$$

*Also, the determinant of each of these matrices is positive and bounded above by unity, and it is equal to unity if, and only if, the matrix involved is the zero matrix. Furthermore,*

$$\left|\begin{array}{cc} I_{n_{1}} & P\\ P^{T} & I_{n_{2}} \end{array}\right|=\left|I_{n_{2}}-P^{T}P\right|.$$

## Appendix B. Some Formulae from [22]

The following equations provide the relevant information-theoretic terms with a slight change of notation: $\Sigma_{Z}$ is now Σ, the indices on the vectors run from 1 to 3 rather than from 0 to 2, and Y is replaced by $X_{3}$.

(A1) $$\begin{array}{cc} I(X_{1};X_{3}) & =\frac{1}{2}\log\frac{1}{\left|\begin{array}{c} I_{n_{3}}-Q^{T}Q \end{array}\right|}, \end{array}$$

(A2) $$\begin{array}{cc} I(X_{2};X_{3}) & =\frac{1}{2}\log\frac{1}{\left|\begin{array}{c} I_{n_{3}}-R^{T}R \end{array}\right|}, \end{array}$$

(A3) $$\begin{array}{cc} I(X_{1};X_{3}|X_{2}) & =\frac{1}{2}\log\frac{\left|\begin{array}{c} I_{n_{2}}-P^{T}P \end{array}\right|\left|\begin{array}{c} I_{n_{3}}-R^{T}R \end{array}\right|}{\left|\Sigma \right|}, \end{array}$$

(A4) $$\begin{array}{cc} I(X_{2};X_{3}|X_{1}) & =\frac{1}{2}\log\frac{\left|\begin{array}{c} I_{n_{2}}-P^{T}P \end{array}\right|\left|\begin{array}{c} I_{n_{3}}-Q^{T}Q \end{array}\right|}{\left|\Sigma \right|} \end{array}$$

(A5) $$\begin{array}{cc} I(X_{1},X_{2};X_{3}) & =\frac{1}{2}\log\frac{\left|\begin{array}{c} I_{n_{2}}-P^{T}P \end{array}\right|}{\left|\Sigma \right|} \end{array}$$

(A6) $$\begin{array}{cc} II(X_{1};X_{2};X_{3}) & =I\left(X_{1},X_{2};X_{3}\right)-I\left(X_{1};X_{3}\right)-I\left(X_{2};X_{3}\right) \end{array}$$

(A7) $$\begin{array}{cc} \; & =\frac{1}{2}\log\frac{\left|\begin{array}{c} I_{n_{2}}-P^{T}P \end{array}\right|\left|\begin{array}{c} I_{n_{3}}-Q^{T}Q \end{array}\right|\left|\begin{array}{c} I_{n_{3}}-R^{T}R \end{array}\right|}{\left|\Sigma \right|} \end{array}$$

## Appendix C. Proof of Lemma 1

From [43] (p. 189), the KL divergence is

$$D(f_{1}\left|\right|f_{2})=\frac{1}{2}\left(\log\frac{\left|\Lambda \right|}{\left|\Phi \right|}-m+\mathrm{Tr}\left(\Phi^{-1}\Lambda \right)\right)$$

since the mean vectors are equal. Each of the diagonal blocks in (13) is positive definite, by Lemma A1, and so possesses a positive definite square root [44] (p. 472). We define the positive definite block diagonal matrix

$$D=\mathrm{diag}\left\{\Sigma_{11}^{\frac{1}{2}},\Sigma_{22}^{\frac{1}{2}},\Sigma_{33}^{\frac{1}{2}}\right\}$$

Hence, we may write

(A8) $$\Phi =D\left[\begin{array}{ccc} I_{n_{1}} & P_{12} & P_{13}\\ P_{12}^{T} & I_{n_{2}} & P_{23}\\ P_{13}^{T} & P_{23}^{T} & I_{n_{3}} \end{array}\right]D\;\mathrm{and}\;\Lambda =D\left[\begin{array}{ccc} I_{n_{1}} & L_{12} & L_{13}\\ L_{12}^{T} & I_{n_{2}} & L_{23}\\ L_{13}^{T} & L_{23}^{T} & I_{n_{3}} \end{array}\right]D$$

where

$$P_{12}=\Sigma_{11}^{-\frac{1}{2}}\Sigma_{12}\Sigma_{22}^{-\frac{1}{2}},\;P_{13}=\Sigma_{11}^{-\frac{1}{2}}\Sigma_{13}\Sigma_{33}^{-\frac{1}{2}},\;P_{23}=\Sigma_{22}^{-\frac{1}{2}}\Sigma_{23}\Sigma_{33}^{-\frac{1}{2}}.$$

and

$$L_{12}=\Sigma_{11}^{-\frac{1}{2}}\Lambda_{12}\Sigma_{22}^{-\frac{1}{2}},\;L_{13}=\Sigma_{11}^{-\frac{1}{2}}\Lambda_{13}\Sigma_{33}^{-\frac{1}{2}},\;L_{23}=\Sigma_{22}^{-\frac{1}{2}}\Lambda_{23}\Sigma_{33}^{-\frac{1}{2}}.$$

Therefore, using standard properties of determinants, we have from

$$\left|\Phi |=\right|\Sigma_{11}\left|\right|\Sigma_{22}\left|\right|\Sigma_{33}|\left|\begin{array}{ccc} I_{n_{1}} & P_{12} & P_{13}\\ P_{12}^{T} & I_{n_{2}} & P_{23}\\ P_{13}^{T} & P_{23}^{T} & I_{n_{3}} \end{array}\right|=|\Sigma_{11}\left|\right|\Sigma_{22}\left|\right|\Sigma_{33}\left|\right|\Phi_{1}|.$$

Use of a similar argument provides a similar expression for the determinant of Λ:

$$\left|\Lambda |=\right|\Sigma_{11}\left|\right|\Sigma_{22}\left|\right|\Sigma_{33}\left|\right|\Lambda_{1}|.$$

It follows that

$$\frac{\left|\Lambda \right|}{\left|\Phi \right|}=\frac{|\Lambda_{1}|}{|\Phi_{1}|}.$$

We now consider the trace term. We have from (A8) that

$$\Phi =D\Phi_{1}D,\;\mathrm{and}\;\Lambda =D\Lambda_{1}D$$

and so

$$\Phi^{-1}\Lambda =D^{-1}\Phi_{1}^{-1}D^{-1}D\Lambda_{1}D=D^{-1}\Phi_{1}^{-1}\Lambda D.$$

Since Tr(ABC)=Tr(CBA), for any three conformable matrices, the required result follows.

## Appendix D. Proof of Lemma 2

Making use of Lemma A1(iii), we may write the determinant of the covariance matrix Σ as

(A9) $$\left|\Sigma \right|=\left|\begin{array}{ccc} I_{n_{1}} & P & Q\\ P^{T} & I_{n_{2}} & R\\ Q^{T} & R^{T} & I_{n_{3}} \end{array}\right|=\left|A|\right|I_{n_{3}}-\left[\begin{array}{cc} Q^{T} & R^{T} \end{array}\right]A^{-1}\left[\begin{array}{c} Q\\ R \end{array}\right]|$$

where

(A10) $$A=\left[\begin{array}{cc} I_{n_{1}} & P\\ P^{T} & I_{n_{2}} \end{array}\right]$$

Since

(A11) $$A^{-1}=\left[\begin{array}{cc} I_{n_{1}}+P{\left(I_{n_{2}}-P^{T}P\right)}^{-1}P^{T} & -P{\left(I_{n_{2}}-P^{T}P\right)}^{-1}\\ -{\left(I_{n_{2}}-P^{T}P\right)}^{-1}P^{T} & {\left(I_{n_{2}}-P^{T}P\right)}^{-1} \end{array}\right]$$

it can be shown that

$${\left[\begin{array}{cc} Q & R \end{array}\right]}^{T}A^{-1}\left[\begin{array}{c} Q\\ R \end{array}\right]=Q^{T}Q+{\left(P^{T}Q-R\right)}^{T}{\left(I_{n_{2}}-P^{T}P\right)}^{-1})\left(P^{T}Q-R\right)$$

From Lemma A1(iii), we have that

(A12) $$\left|A|=\right|I_{n_{2}}-P^{T}P|$$

and putting these results together gives

(A13) $$\left|\Sigma |=\right|I_{n_{2}}-P^{T}P||I_{n_{3}}-Q^{T}Q-{\left(P^{T}Q-R\right)}^{T}{\left(I_{n_{2}}-P^{T}P\right)}^{-1}\left(P^{T}Q-R\right)|.$$

We now apply this result to obtain expressions for the determinants of $\Sigma_{5}$ and $\Sigma_{6}$, which are defined in Table 1. In $\Sigma_{5}$, *R* is replaced by $P^{T}Q$ and making this substitution in (A13) gives

(A14) $$|\Sigma_{5}\left|=\right|I_{n_{2}}-P^{T}P||I_{n_{2}}-Q^{T}Q|.$$

Similarly, replacing *Q* by PR in (A13), gives the determinant of $\Sigma_{6}$, after some manipulation, as

(A15) $$|\Sigma_{6}\left|=\right|I_{n_{2}}-P^{T}P||I_{n_{2}}-R^{T}R|.$$

For the final result, we have that

$$\Sigma_{5}-\Sigma_{6}=\left[\begin{array}{ccc} 0 & 0 & Q-PR\\ 0 & 0 & P^{T}Q-R\\ Q^{T}-R^{T}P^{T} & Q^{T}P-R^{T} & 0 \end{array}\right]$$

from which it is clear that Q=0,R=0 implies that $\Sigma_{5}=\Sigma_{6}$. Conversely,

$$\begin{array}{cc} \Sigma_{5}=\Sigma_{6} & \Rightarrow Q=PR\;\;\mathrm{and}\;\;R=P^{T}Q\\ \; & \Rightarrow \left(I_{n_{2}}-P^{T}P\right)R=0\;\;\mathrm{and}\;\;\left(I_{n_{1}}-PP^{T}\right)Q=0\\ \; & \Rightarrow Q=0\;\;\mathrm{and}\;\;R=0 \end{array}$$

by Lemma A2. For the last equivalence, we note by (A13) that Q=0 and R=0 implies that $I(X_{1},X_{2};X_{3})=0$. Conversely, by (A4) and (A13) and the non-negativity of mutual information, $I(X_{1},X_{2};X_{3})=0$ implies that $I(X_{1};X_{3})=0$ and $I(X_{2};X_{3})=0$, which imply from (A1) and (A2) and Lemma A2 that Q=0 and R=0.

## Appendix E. Proof of Lemma 3

$\Sigma_{t}$ and $\Sigma_{t}^{\prime }$ are each positive definite if and only their inverses are positive definite. We work with the inverses. When t=0 or t=1 clearly both inverses are positive definite, since the inverses of $\Sigma_{5}$ and $\Sigma_{6}$ are positive definite. Suppose that 0<t<1 and consider for $\Sigma_{t}^{-1}$, and for any $x\in R^{m}$, the quadratic form

$$x^{T}\left(\left(1-t\right)\Sigma_{5}^{-1}+t\Sigma_{6}^{-1}\right)x=\left(1-t\right)x^{T}\Sigma_{5}^{-1}x+tx^{T}\Sigma_{6}^{-1}x.$$

This term is non-negative for every $x\in R^{m}$ since the inverses of $\Sigma_{5}$ and $\Sigma_{6}$ are positive definite and 0<t<1. Clearly, for the same reasons, this quadratic form is equal to zero if and only if x is the zero vector in $R^{m}$, hence the result. A similar argument shows that $\Sigma_{t}^{\prime \;-1}$ is positive definite when 0<t<1.

For t∈F, we know that the matrix $\Sigma_{t}^{-1}=\left(1-t\right)\Sigma_{5}^{-1}+t\Sigma_{6}^{-1}$ is positive definite. The matrix $\Sigma_{t}^{\prime \;-1}=\left(1-t\right)\Sigma_{6}+t\Sigma_{5}$ may be written in two ways as

$$\left(1-t\right)\Sigma_{6}+t\Sigma_{5}=\Sigma_{6}\left(\left(1-t\right)\Sigma_{5}^{-1}+t\Sigma_{6}^{-1}\right)\Sigma_{5}=\Sigma_{5}\left(\left(1-t\right)\Sigma_{5}^{-1}+t\Sigma_{6}^{-1}\right)\Sigma_{6}.$$

which is a product of three positive definite matrices. Since the last two expressions are transposes of each other it follows that this product is symmetric. Hence, from a result by E. P. Wigner [45], we deduce that $\Sigma_{t}^{\prime \;-1}$ is positive definite, so that t∈G. A similar argument shows that t∈G⇒t∈F. Thus, we have proved that the feasible sets *F* and *G* are equal.

## Appendix F. Proof of Lemma 4

For the first part, we consider (3):

(A16) $$\Sigma_{t}^{-1}\Sigma =\left(1-t\right)\Sigma_{5}^{-1}\Sigma +t\Sigma_{6}^{-1}\Sigma$$

and apply the trace operator, which is linear, to obtain

(A17) $$\mathrm{Tr}\left(\Sigma_{t}^{-1}\Sigma \right)=\left(1-t\right)\mathrm{Tr}\left(\Sigma_{5}^{-1}\Sigma \right)+t\mathrm{Tr}\left(\Sigma_{6}^{-1}\Sigma \right).$$

Consider $\mathrm{Tr}(\Sigma_{5}^{-1}\Sigma ).$ We may write this as

(A18) $$\mathrm{Tr}\left(\Sigma_{5}^{-1}\Sigma \right).=\mathrm{Tr}(\Sigma_{5}^{-1}(\Sigma_{5}+\left(\Sigma -\Sigma_{5}\right)=\mathrm{Tr}\left(I_{m}\right)+\mathrm{Tr}\left(\Sigma_{5}^{-1}\left(\Sigma -\Sigma_{5}\right)\right)$$

Now, from (12) and the form of $\Sigma_{5}$ in Table 1, we have that

$$\Sigma -\Sigma_{5}=\left[\begin{array}{ccc} 0 & 0 & 0\\ 0 & 0 & R-P^{T}Q\\ 0 & R^{T}-Q^{T}P & 0 \end{array}\right].$$

The pdf $p_{5}$ is defined by the constraint $K_{23}=0$ in the inverse covariance matrix *K*. Hence, $\Sigma_{5}^{-1}$ has the form

$$\Sigma_{5}^{-1}=\left[\begin{array}{ccc} {}* & * & *\\ {}* & * & 0\\ {}* & 0 & * \end{array}\right]$$

Performing the multiplication of these block matrices shows that the diagonal blocks of $\Sigma_{5}^{-1}\left(\Sigma -\Sigma_{5}\right)$ are all equal to a zero matrix, and so have trace equal to zero. Hence, the trace of this matrix is equal to zero. Since $\mathrm{Tr}(I_{m})=m$, it follows from (A18) that this matrix has trace equal to *m*.

Now consider the trace of $\Sigma_{6}^{-1}\Sigma$. From (12) and the form of $\Sigma_{6}$ in Table 1, we have that

$$\Sigma -\Sigma_{6}=\left[\begin{array}{ccc} 0 & 0 & Q-PR\\ 0 & 0 & 0\\ Q^{T}-R^{T}P^{T} & 0 & 0 \end{array}\right].$$

The pdf $p_{6}$ is defined by the constraint $K_{13}=0$ in the inverse covariance matrix *K*. Hence, $\Sigma_{6}^{-1}$ has the form

$$\Sigma_{6}^{-1}=\left[\begin{array}{ccc} {}* & * & 0\\ {}* & * & *\\ 0 & * & * \end{array}\right]$$

By adopting a similar argument to that given above for $\Sigma_{5}$, it follows that $\mathrm{Tr}(\Sigma_{6}^{-1}\Sigma )=m$. The required result follows from (A17). Now for the second part of the proof, we use Lemma 1 and the trace result just derived to write

(A19) $$D(p||p_{t})=\frac{1}{2}\log\frac{|\Sigma_{t}|}{\left|\Sigma \right|}-\frac{1}{2}m+\frac{1}{2}\mathrm{Tr}\left(\Sigma_{t}^{-1}\Sigma \right)=\frac{1}{2}\log\frac{|\Sigma_{t}|}{\left|\Sigma \right|}.$$

$\Sigma_{t}^{-1}$ may be written as

(A20) $$\Sigma_{t}^{-1}=\Sigma_{5}^{-1}\left(\left(1-t\right)\Sigma_{6}+t\Sigma_{5}\right)\Sigma_{6}^{-1}$$

and so, by Lemma 2, we have

(A21) $$|\Sigma_{t}|=\frac{|\Sigma_{5}\left|\right|\Sigma_{6}|}{|\left(1-t\right)\Sigma_{6}+t\Sigma_{5}|}=\frac{|I_{n_{2}}-P^{T}{P|}^{2}|I_{n_{3}}-Q^{T}Q||I_{n_{3}}-R^{T}R|}{|\left(1-t\right)\Sigma_{6}+t\Sigma_{5}|}$$

Hence

(A22) $$g\left(t\right)=\frac{1}{2}\log\frac{|I_{n_{2}}-P^{T}{P|}^{2}|I_{n_{3}}-Q^{T}Q||I_{n_{3}}-R^{T}R|}{\left|\Sigma \right|}-\frac{1}{2}\log\left|\left(1-t\right)\Sigma_{6}+t\Sigma_{5}\right|$$

We wish to minimize g(t) with respect to *t* under the constraint that $t\in F^{\circ }$. We set $H\left(t\right)=\left(1-t\right)\Sigma_{6}+t\Sigma_{5}$, which is positive definite by Lemma 3, and apply Jacobi’s formula and the chain rule. Differentiating (A22) with respect to *t* we obtain

$$\begin{array}{cc} g^{\prime }\left(t\right) & =-\frac{1}{2}\frac{1}{\left|H(t)\right|}\times \left|H\left(t\right)\right|\times \mathrm{Tr}\left(H{\left(t\right)}^{-1}\left(\Sigma_{5}-\Sigma_{6}\right)\right)\\ \; & =-\frac{1}{2}\mathrm{Tr}\left(H{\left(t\right)}^{-1}\left(\Sigma_{5}-\Sigma_{6}\right)\right) \end{array}$$

Further differentiation yields

$$\begin{array}{cc} g^{\prime \prime }\left(t\right) & =-\frac{1}{2}\mathrm{Tr}\left(H{\left(t\right)}^{-1}\left(\Sigma_{5}-\Sigma_{6}\right)H{\left(t\right)}^{-1}\left(\Sigma_{5}-\Sigma_{6}\right)\right)\\ & =-\frac{1}{2}\mathrm{Tr}\left({\left[H{\left(t\right)}^{-1}\left(\Sigma_{5}-\Sigma_{6}\right)\right]}^{2}\right)=-\frac{1}{2}\sum_{i=1}^{m}\lambda_{i}^{2}, \end{array}$$

where the $\lambda_{i}$ are the eigenvalues of the matrix $H{\left(t\right)}^{-1}\left(\Sigma_{5}-\Sigma_{6}\right)$. Since H(t) is positive definite it possesses a positive definite square root K(t), say. Since the eigenvalues of $H{\left(t\right)}^{-1}\left(\Sigma_{5}-\Sigma_{6}\right)$ are the same as those of $K\left(t\right)\left(\Sigma_{5}-\Sigma_{6}\right)K\left(t\right)$, which is symmetric and so has real eigenvalues, and since the joint mutual information is positive (and so by Lemma 2 $\Sigma_{5}\neq \Sigma_{6}$), it follows that at least one of the eigenvalues must be non-zero. Hence, $g^{\prime \prime }\left(t\right)<0,\forall t\in F^{\circ }$. Therefore, g(t) is strictly convex on the convex set $F^{\circ }$ and the minimum is unique, and given by the value $t^{*}$ which satisfies the necessary condition $g^{\prime }\left(t\right)=0$. (This $t^{*}$ turns out to be exactly the value of *t* that is required for the two forms of shared information to be equal.) Of course, the minimum could occur at an endpoint of the interval *F*, in which case the minimum value of g(t) is the value taken at the endpoint.

Finally, we write

(A23) $$|\left(1-t\right)\Sigma_{6}+t\Sigma_{5}|=\left|\begin{array}{cc} A & B(t)\\ B{\left(t\right)}^{T} & I_{n_{3}} \end{array}\right|=\left|A|\right|I_{n_{3}}-B{\left(t\right)}^{T}A^{-1}B\left(t\right)|,$$

where *A* is defined in (A10), with inverse given by (A11), and

$$B\left(t\right)=\left[\begin{array}{c} (1-t)Q+tPR\\ \left(1-t\right)P^{T}Q+tR. \end{array}\right]$$

Some matrix calculation then gives

$$A^{-1}B\left(t\right)=\left[\begin{array}{c} (1-t)Q\\ tR \end{array}\right]$$

and also

(A24) $$B{\left(t\right)}^{T}A^{-1}B\left(t\right)={\left(1-t\right)}^{2}Q^{T}Q+t\left(1-t\right)\left(R^{T}P^{T}Q+Q^{T}PR\right)+t^{2}R^{T}R.$$

Now from (A12) and (A23) it follows that $|\left(1-t\right)\Sigma_{6}+t\Sigma_{5}|$ is equal to

(A25) $$|I_{n_{2}}-P^{T}P|\left|\begin{array}{c} I_{n_{3}}-{\left(1-t\right)}^{2}Q^{T}Q-t\left(1-t\right)\left(R^{T}P^{T}Q+Q^{T}PR\right)-t^{2}R^{T}R \end{array}\right|$$

Making use of this equation and also (A22), and replacing *t* by the optimal $t^{*}$, we have that the minimum of g(t), when $t^{*}\in F$, is

(A26) $$g\left(t^{*}\right)=\frac{1}{2}\log\frac{|I_{n_{2}}-P^{T}P||I_{n_{3}}-Q^{T}Q||I_{n_{3}}-R^{T}R|}{\left|\Sigma \right|}+\frac{1}{2}\log\frac{1}{d(t^{*})},$$

where

(A27) $$d\left(t\right)=\left|\begin{array}{c} I_{n_{3}}-{\left(1-t\right)}^{2}Q^{T}Q-t\left(1-t\right)\left(R^{T}P^{T}Q+Q^{T}PR\right)-t^{2}R^{T}R \end{array}\right|.$$

## Appendix G. Proof of Lemma 5

From (4) we know that

(A28) $$D(p||p_{5})=D(p||p_{t*})+D(p_{t^{*}}\left|\right|p_{5}).$$

Then, by Lemmas 1 and 4,

$$D(p||p_{5})=\frac{1}{2}\log\frac{|\Sigma_{5}|}{\left|\Sigma \right|}-\frac{1}{2}m+\frac{1}{2}\mathrm{Tr}\left(\Sigma_{5}^{-1}\Sigma \right)=\frac{1}{2}\log\frac{|\Sigma_{5}|}{\left|\Sigma \right|}.$$

Also,

$$D(p_{t^{*}}\left|\right|p_{5})=\frac{1}{2}\log\frac{|\Sigma_{5}|}{|\Sigma_{t^{*}}|}-\frac{1}{2}m+\frac{1}{2}\mathrm{Tr}\left(\Sigma_{5}^{-1}\Sigma_{t^{*}}\right)$$

and

$$D(p||p_{t^{*}})=\frac{1}{2}\log\frac{|\Sigma_{t^{*}}|}{\left|\Sigma \right|}$$

by (20) with $t=t^{*}$. By substituting these expressions into (A28) it follows that $\mathrm{Tr}(\Sigma_{5}^{-1}\Sigma_{t^{*}})\;=\;m$. The proof that $\mathrm{Tr}(\Sigma_{6}^{-1}\Sigma_{t^{*}})=m$ is obtained by using a very similar argument, starting with (5).

## Appendix H. Proof of Proposition 1

The formula for synergy is the minimum value of g(t) as provided in (A26). From (30), Lemmas 1 and 5, we have that

(A29) $$\begin{array}{cc} \mathrm{Unq}1 & =\frac{1}{2}\log\frac{|\Sigma_{6}|}{|\Sigma_{t^{*}}|}\\ \; & =\frac{1}{2}\log\frac{|\Sigma_{5}\left|\right|\Sigma_{6}|}{|\left(1-t\right)\Sigma_{6}+t\Sigma_{5}|},\;\mathrm{by}\;\left(A21\right),\\ \; & =\frac{1}{2}\log\frac{|I_{n_{2}}-P^{T}P|d\left(t^{*}\right)}{|I_{n_{2}}-P^{T}P||I_{n_{3}}-Q^{T}Q|},\;\mathrm{by}\;\left(A25\right)\;\mathrm{and}\;\left(A27\right)\;\mathrm{and}\;\mathrm{Lemma}\;2\;,\\ \; & =\frac{1}{2}\log\frac{1}{|I_{n_{3}}-Q^{T}Q|}-\frac{1}{2}\log\frac{1}{d(t^{*})} \end{array}$$

By making use of a similar calculation we find that

(A30) $$\mathrm{Unq}2=\frac{1}{2}\log\frac{|\Sigma_{5}|}{|\Sigma_{t^{*}}|}=\frac{1}{2}\log\frac{1}{|I_{n_{3}}-R^{T}R|}-\frac{1}{2}\log\frac{1}{d(t^{*})}$$

The two expressions for the shared information are

$$I\left(X_{1};X_{3}\right)-\mathrm{Unq}1\;\mathrm{or}\;I\left(X_{2};X_{3}\right)-\mathrm{Unq}2.$$

By using (6), (7), (A29) and (A30) it is easy to see that both expressions for the shared information, Shd, are equal to $\frac{1}{2}\log\frac{1}{d(t^{*})}.$

## Appendix I. Proof of Proposition 2

### Appendix I.1. Non-Negativity

Since the Syn, Unq1 and Unq2 components in the $I_{\mathrm{ig}}$ PID have been defined to be KL divergences they are non-negative. It remains to show that Shd is non-negative. From Appendix H, we see that this is the case if and only if $0<d(t^{*})<=1$. From (A23), (A24) and (A27) we have, with $t=t^{*}$, that

$$|\left(1-t^{*}\right)\Sigma_{6}+t^{*}\Sigma_{5}|=\left|\begin{array}{cc} A & B(t^{*})\\ B{\left(t^{*}\right)}^{T} & I_{n_{3}} \end{array}\right|=\left|A|\right|I_{n_{3}}-B{\left(t^{*}\right)}^{T}A^{-1}B\left(t^{*}\right)|,\;$$

and that

$$d\left(t^{*}\right)=\left|I_{n_{3}}-B{\left(t^{*}\right)}^{T}A^{-1}B\left(t^{*}\right)\right|$$

Since $t^{*}\in F$, we know by Lemma 3 that $t^{*}\in G$, and so this matrix is positive definite. It follows from Lemma A1(iii) that $I_{n_{3}}-B{\left(t^{*}\right)}^{T}A^{-1}B\left(t^{*}\right)$ is positive definite and from Lemma A1(i) that *A* is positive definite, as is $A^{-1}$. Therefore, $A^{-1}$ possesses a (symmetric) positive definite square root, *C* say, and we may write

$$I_{n_{3}}-B{\left(t^{*}\right)}^{T}A^{-1}B\left(t^{*}\right)=I_{n_{3}}-X^{T}X,\;\mathrm{with}\;X=CB\left(t^{*}\right).$$

Then the matrix $X^{T}X$ is positive semi-definite and so has non-negative eigenvalues, $\lambda_{1},\lambda_{2},\ldots \lambda_{n_{3}}$. We denote the eigenvalues of $I_{n_{3}}-X^{T}X$ as $\left\{1-\lambda_{i}:i=1,2,...,n_{3}\right\}$. Since $I_{n_{3}}-X^{T}X$ is positive definite we know that $1-\lambda_{i}>0$ for $i=1,2,...,n_{3}.$ It follows that $0<1-\lambda_{i}\leq 1$ for $i=1,2,...,n_{3}.$ Since the determinant of a square matrix is the product of its eigenvalues we have that

$$d\left(t^{*}\right)\equiv \left|I_{n_{3}}-X^{T}X\right|=\prod_{i=1}^{n_{3}}\left(1-\lambda_{i}\right),$$

and so $0<d(t^{*})\leq 1$, hence the result.

### Appendix I.2. Self-Redundancy

The property of self-redundancy considers the case of $X_{1}=X_{2}$, i.e., both sources are the same, and it requires us to show that

$$\mathrm{Shd}\equiv I_{shd}\left(X_{1},X_{1};X_{3}\right]=I\left[X_{1};X_{3}\right].$$

When the sources are the same we have $n_{1}=n_{2},Q=R$ and $P=I_{n_{1}}$, which results in a singular covariance matrix. Therefore, we take $P=\left(1-\epsilon \right)I_{n_{1}}$, for very small ϵ such that 0<ϵ<1, and let ϵ→0+. Using this information in (A27) we have that

$$\begin{array}{cc} d(t^{*}) & =|I_{n_{3}}-{\left(1-t^{*}\right)}^{2}Q^{T}Q-\left(1-\epsilon \right)t^{*}\left(1-t^{*}\right)Q^{T}Q-t^{*2}Q^{T}Q|\\ \; & =|I_{n_{3}}-Q^{T}Q+\epsilon t^{*}\left(1-t^{*}\right)Q^{T}Q|\\ \; & \to |I_{n_{3}}-Q^{T}Q|,\;\mathrm{as}\;\epsilon \to 0+. \end{array}$$

Therefore $\mathrm{Shd}\to \frac{1}{2}\log\frac{1}{|I_{n_{3}}-Q^{T}Q|}$, which is equal to $I[X_{1};X_{3}]$ by (A1).

Out of interest, it seems worthwhile to check the limits for the classical information measures given in (A3)–(A7). From (A3) and (A13), after some cancellation, we have that

$$\begin{array}{cc} I[X_{1};X_{3}|X_{2}] & =\frac{1}{2}\log\frac{|I_{n_{3}}-Q^{T}Q|}{|I_{n_{3}}-Q^{T}Q-{\left(P^{T}Q-R\right)}^{T}{\left(I_{n_{2}}-P^{T}P\right)}^{-1}\left(P^{T}Q-R\right)|}\\ \; & =\frac{1}{2}\log\frac{|I_{n_{3}}-Q^{T}Q|}{|I_{n_{3}}-Q^{T}Q+\frac{\epsilon }{2-\epsilon }Q^{T}Q|}\\ \; & \to 0,\;\mathrm{as}\;\epsilon \to 0+. \end{array}$$

Also, since Q=R,

$$\begin{array}{cc} I[X_{2};X_{3}|X_{1}] & =\frac{1}{2}\log\frac{|I_{n_{3}}-R^{T}R|}{|I_{n_{3}}-R^{T}R+\frac{\epsilon }{2-\epsilon }R^{T}R|}\\ \; & \to 0,\;\mathrm{as}\;\epsilon \to 0+. \end{array}$$

Similarly, for the joint mutual information and the interaction information:

$$I\left[X_{1},X_{2};X_{3}\right]\to \frac{1}{2}\log\frac{1}{|I_{n_{3}}-Q^{T}Q|},$$

and

$$I\left[X_{1};X_{2};X_{3}\right]\to -\frac{1}{2}\log\frac{1}{|I_{n_{3}}-Q^{T}Q|,}$$

as expected, since ϵ→0+.

### Appendix I.3. Symmetry

To validate the symmetry property we require to prove that $I_{shd}\left[X_{1},X_{2};X_{3}\right]$ is equal to $I_{shd}\left[X_{2},X_{1};X_{3}\right]$. Swapping $X_{1}$ and $X_{2}$ means that the sources are now in order $X_{2},X_{1},X_{3}$ and the covariance matrix in (12) becomes

$$\left[\begin{array}{ccc} I_{n_{2}} & P^{T} & R\\ P & I_{n_{1}} & Q\\ R^{T} & Q^{T} & I_{n_{3}} \end{array}\right],$$

The switching of $X_{1}$ and $X_{2}$ means also that the probability distributions on $S_{5}$ and $S_{6}$ swap, since

$$\begin{array}{cc} S_{5}:\;p\left(x_{2}|x_{1}\right)p\left(x_{3}|x_{1}\right)p\left(x_{1}\right) & ⟶p\left(x_{1}|x_{2}\right)p\left(x_{3}|x_{2}\right)p\left(x_{2}\right)\\ S_{6}:\;p\left(x_{1}|x_{2}\right)p\left(x_{3}|x_{2}\right)p\left(x_{2}\right) & ⟶p\left(x_{2}|x_{1}\right)p\left(x_{3}|x_{1}\right)p\left(x_{1}\right) \end{array}$$

and the corresponding covariance matrices are

$$\Sigma_{5}^{\prime }=\left[\begin{array}{ccc} I_{n_{2}} & P^{T} & R\\ P & I_{n_{1}} & PR\\ R^{T} & R^{T}P^{T} & I_{n_{3}} \end{array}\right]\;\mathrm{and}\;\Sigma_{6}^{\prime }=\left[\begin{array}{ccc} I_{n_{2}} & P^{T} & P^{T}Q\\ P & I_{n_{1}} & Q\\ Q^{T}P^{T} & Q^{T} & I_{n_{3}} \end{array}\right]$$

We now apply a similar argument to that used in the proof of Lemma 4.

$$|\left(1-t\right)\Sigma_{6}^{\prime }+t\Sigma_{5}^{\prime }|=\left|\begin{array}{cc} A^{\prime } & B{\left(t\right)}^{\prime }\\ B{\left(t\right)}^{\prime T} & I_{n_{3}} \end{array}\right|=\left|A|\right|I_{n_{3}}-B{\left(t\right)}^{\prime T}A^{\prime -1}B{\left(t\right)}^{\prime }|,$$

where $A^{\prime }$ and $A^{\prime -1}$ are

$$A^{\prime }=\left[\begin{array}{cc} I_{n_{2}} & P^{T}\\ P & I_{n_{1}} \end{array}\right]$$

$$A^{\prime -1}=\left[\begin{array}{cc} I_{n_{2}}+P^{T}{\left(I_{n_{2}}-PP^{T}\right)}^{-1}P & -P^{T}{\left(I_{n_{2}}-PP^{T}\right)}^{-1}\\ -{\left(I_{n_{2}}-PP^{T}\right)}^{-1}P & {\left(I_{n_{2}}-PP^{T}\right)}^{-1} \end{array}\right]$$

and

$$B^{\prime }\left(t\right)=\left[\begin{array}{c} \left(1-t\right)P^{T}Q+tR\\ (1-t)Q+tPR \end{array}\right].$$

Some matrix calculation then gives

$$A^{\prime -1}B^{\prime }\left(t\right)=\left[\begin{array}{c} tR\\ (1-t)Q \end{array}\right]$$

and also

$$B{\left(t\right)}^{\prime T}A^{\prime -1}B^{\prime }\left(t\right)={\left(1-t\right)}^{2}Q^{T}Q+t\left(1-t\right)\left(R^{T}P^{T}Q+Q^{T}PR\right)+t^{2}R^{T}R.$$

Hence,

$$d^{\prime }\left(t\right)=\left|\begin{array}{c} I_{n_{3}}-{\left(1-t\right)}^{2}Q^{T}Q-t\left(1-t\right)\left(R^{T}P^{T}Q+Q^{T}PR\right)-t^{2}R^{T}R \end{array}\right|$$

which is identical to the expression of d(t) obtained in (A27). It follows that $d\left(t^{*}\right)=d^{\prime }\left(t^{*}\right)$ and that the shared information is unchanged by swapping $X_{1}$ and $X_{2}$.

### Appendix I.4. Monotonicity on the Redundancy Lattice

We use the term ‘redundancy’ here for convenience rather than ‘shared information’, since both terms mean exactly the same thing. A redundancy lattice is defined in [1]. When there are two sources and a target, there are four terms of interest that are usually denoted by {{1}{2},{1},{2},{12}}, which are the terms in the redundancy lattice. For monotonicity it is required that the redundancy values for these four terms satisfy the inequalities {1}{2}≤{1}≤{12} and that {1}{2}≤{2}≤{12}.

The redundancy value for term {12} is the self-redundancy $I_{shd}\left[\left(X_{1},X_{2}\right),\left(X_{1},X_{2}\right);X_{3}\right]$ which is, by the self-redundancy property, equal to the joint mutual information $I[X_{1},X_{2};X_{3}]$. Similarly, the redundancy values for the terms {1} and {2} are, by self-redundancy, the mutual information $I[X_{1};X_{3}]$ and $I[X_{2};X_{3}]$, respectively. The final term {1}{2} is equal to the shared information defined in Proposition 1.

Since the PID defined in Proposition 1 possesses the non-negativity property it follows from (6)–(10) that the redundancy measure is monotonic on the redundancy lattice.

## Appendix J. Computation

Code was written using R [46] in RStudio [46] to compute the $I_{\mathrm{ig}}$ PID. This code together with R code to compute the $I_{\mathrm{dep}}$ and $I_{\mathrm{mmi}}$ PIDs is available from https://github.com/JWKay/PID (accessed on 17 June 2024). The $I_{\mathrm{ig}}$ code first checks whether the input covariance matrix is positive-definite. If so, the feasible region *F* is computed by defining a function whose value indicates whether or not the matrix $\Sigma_{t}$ is positive definite, and then applying the uniroot root-finding algorithm. The constrained optimisation is performed by using the base R function optim. The code produces a plot of g(t) and returns the numerical results. Details of the pre-processing employed to make use of the $I_{\mathrm{ig}}$ formulae presented in Proposition 1 are the same as used with the $I_{\mathrm{dep}}$ PID and are available from Appendix D [22].
